# Serum and brain natural copper stable isotopes in a mouse model of Alzheimer’s disease

**DOI:** 10.1038/s41598-019-47790-5

**Published:** 2019-08-15

**Authors:** Frédéric Moynier, John Creech, Jessica Dallas, Marie Le Borgne

**Affiliations:** 1 Institut de Physique du Globe de Paris, Université de Paris, CNRS 7154, 1 rue Jussieu, 75238 Paris, cedex 05 France; 2 Institut Universitaire de France, 75005 Paris, France; 3 Université de Paris, LVTS, Inserm U1148, F-75018, Paris, France; 4 Département Hospitalo-Universitaire DHU FIRE, Paris, France

**Keywords:** Biogeochemistry, Geochemistry

## Abstract

Alzheimer’s disease is associated with the production of Cu rich aβ fibrils. Because monitoring the changes in Cu level of organs has been proposed to follow the evolution of the disease, we analyzed the copper isotopic composition of serum and brain of APPswe/PSEN1dE9 transgenic mice, a model of Alzheimer’s disease, and wild-type (WT) controls. Serum composition of 3, 6, 9 and 12-month-old mice, as well as the composition of 9 brains of 12-month-old mice are reported. In WT mice, brains were ~1‰ isotopically heavier than serum, and the Cu isotopic composition of the serum was isotopically different between males and females. We propose that this effect of sex on the Cu isotopic budget of the serum may be related to a difference of Cu speciation and relative abundance of Cu carriers. Brains of APPswe/PSEN1dE9 mice were slightly lighter than brains of WT mice, while not statistically significant. This trend may reflect an increase of Cu(I) associated with the formation of Aβ fibrils. The Cu isotopic composition of the brains and serum were correlated, implying copper transport between these two reservoirs, in particular a transfer of Cu(I) from the brain to the serum. Altogether, these data suggest that Cu stable isotopic composition of body fluid may have the potential to be used as detection tools for the formation of Aβ fibrils in the brain, but further work has to be done.

## Introduction

Alzheimer’s disease (AD) is one of the main causes of death in high-income countries and the fifth leading cause of death of US citizen over 65^1^. It is characterized by a loss of brain function affecting reasoning, memory and comportment. The major physiological features of AD are the formation of neurofibrillary tangles by neuronal accumulations of abnormal hyperphosphorylated tau filaments and the formation of senile plaques by extracellular deposits of amyloid β (Aβ) fibrils, mostly the 1 to 42 peptide (Aβ_1-42_). Early diagnosis of AD is a major challenge and current work focuses on the detection of excess of total tau and Aβ in cerebrospinal fluid, and on using imaging of the brain by positron emission tomography and magnetic resonance imaging (MRI) for Aβ plaques (e.g. refs^2–4^).

The homeostasis of metals, such as Fe, Zn, Al or Cu, is modified in patients with AD (e.g. ref.^5^). These metals are concentrated in the Aβ plaques and in consequences in the neocortex of AD patients (e.g. refs^6,7^). The changes in metal homeostatis in AD have been tested as a diagnosis tool as the concentrations in the Aβ plaques should impact the concentration in other organs and fluids (e.g. red blood cells and serum). However, studies focussing on elemental abundance measurements in the serum of AD patients are inconsistent, showing overall Zn abundance decreasing^5^, increasing^8^ or unchanged^9^. Elemental abundance variations in the serum may be affected by factors unrelated to AD, such as intestinal absorption of Zn^10^, which greatly complicates the interpretation of this data for AD diagnosis.

We and others recently showed that the natural stable isotopic composition of metals such as Zn, Cu and Fe naturally varies between body organs^11–13^. In particular, blood (red blood cells and/or serum) are isotopically distinct in Zn, Cu and Fe from the brain^11–13^). Isotopic fractionation is due to the difference of bonding environments of the elements between different organs^12^. For example, in normal conditions, Zn is preferentially bound to cysteine-rich protein (metallothionein) in the brain, which preferentially concentrates light isotopes, while in red blood cells (RBCs) Zn is bound to histidine-rich proteins (carbonic anhydrase) that concentrate heavy isotopes^12,13^. On the other hand, in Aβ plaques Zn is bonded to isotopically heavy amino-acids (histidine and glutamate)^14^ and therefore should be isotopically heavier in AD patients. Following this logic, Moynier *et al*.^15^ studied the Zn isotopic composition of brain and blood samples during the development of transgenic APPswe/PSEN1dE9 and wild type controls mice and found that, as predicted, Zn was isotopically heavier in the brain of AD mice than in age-matched wild types. However, the isotopic variations among serum samples were not large enough to allow for the detection of an isotopic effect.

While Zn is only present under one redox state (Zn^2+^), Cu is present in both 1+ and 2+ forms. The presence of redox variations of Cu potentially enhances the magnitude of isotopic variations compared to Zn (e.g. see ab initio calculations for various Cu(I) and Cu(II) species in ref.^16^). In addition, Balter *et al*.^13^ showed that Cu is isotopically heavier in the brain of mice than in the whole blood (by about 1 permil for the ^65^Cu/^63^Cu ratio) suggesting that, as done with Zn isotopes^15^, Cu isotopes could be used as tracers of metal dyshomeostasis in the brain. This potential has been confirmed by the discovery of Cu isotopic fractionation in the cerebrospinal fluid of patients with amyotrophic lateral sclerosis compared to control patients^17^.

In the case of AD, Cu participates in the structure of the Aβ plaques; Cu is enriched in the plaques in mice (e.g. ref.^18^) and humans^19^ compared to normal adjacent brain zones. Copper bound to the Aβ plaques could be formed under both +I and +II form^20,21^. In the brain copper is principally stored in proteins under its reduced form Cu(I) binded to metallothionein and glutathione both cystetine-rich proteins^22,23^ and a fraction of Cu(II) is present in synapses^24^. The accumulation of Cu(II) in Aβ plaques changes the speciation of Cu in the brain in the brain which may affect Cu in body fluids such as blood, as suggested from the global Cu level increase observed in the serum of AD patients^25^. This change may be traceable with Cu stable isotopic composition. Furthermore, formation of Aβ plaques affect the global redox state of Cu in the brain, and can lead to the increase of reactive oxygen species, oxidative stress and loss of cognitive functions (e.g. ref.^26^).

Here we tested whether Cu stable isotopic composition of serum and brain are modified by the formation of Aβ plaques in mice with late stage AD. We report the Cu isotopic composition of the serum from 23 transgenic APPswe/PSEN1dE9^27,28^ and wild type (WT) mice at various stages (3 month, 6 month, 9 month and 12 month). In addition, we report the Cu isotopic composition of the brains of 9 of the 12-month mice.

## Method

### Ethical statement

All measurements reported here are analyses of existing samples collected under a protocol approved by the Washington University Animal Studies Committee (ASC). The Procedures were approved by the Washington University Animal Studies Committee (ASC), (Protocol #20120013).

### Mice

The animals used for this study correspond to some of the mice reported in Moynier *et al*.^15^, for which we still had samples after the analysis of Zn isotopic composition: APPswe/PSEN1dE9 (AD) and wild-type (WT) littermate controls on a C57BL/6 and C3H mixed background. At 3, 6, 9 and 12 months, blood samples were collected. After 12 months, the mice were killed and their brains were collected. All mice were housed under specific pathogen-free conditions in the Washington University animal facilities in accordance with institutional guidelines, and were given the same diet from birth. The diet is the one reported in Moynier *et al*.^15^. AD mice were originally obtained from The Jackson Laboratory, then bred and housed in the Washington University animal facilities by crossing to B3C3F1/J (from The Jackson Laboratory).

### Sample collection

Samples preparation was described in Moynier *et al*.^15^. Mice were anesthetized with ketamine and xylazine. Anesthesia was assessed by the toe pinch method. The thoracic cage was opened and blood was collected in polypropylene microtubes by a cardiac puncture. The vena cava was sectioned under the kidneys, and PBS was injected in the left ventricle for organ perfusion. Death was assessed by cervical dislocation. Serum was separated from red blood cells by centrifugation. After blood collection, mice were perfused by injecting phosphate buffered saline (PBS) through the heart with a dissected hepatic vein to remove blood from organs. Brains were harvested using instruments in stainless steel. All samples were stored in polypropylene microtubes or cryogenic vials (Corning). Harvested organs were snap-frozen and kept frozen until dissolution.

### Chemical purification and mass-spectrometry

The Cu purification and isotopic measurements were performed at the Institut de Physique du Globe de Paris (IPGP). The cleaning and dissolution of the samples is similar as the one used previously for Zn isotopic measurements^15^. Brain were cleaned twice using 18.2 MΩ cm water and the brain and serum were dissolved using a mixture of HNO_3_ (4 times sub-boiled) and Seastar© optima grade H_2_O_2_ in closed Teflon beakers for one to two days. This method provides a complete dissolution of the organs with minimum contamination (as opposed to the combustion of the organs in an oven).

Copper is purified by ion exchange chromatography following a procedure adapted from Maréchal *et al*.^29^ and similar to what we used recently^30,31^. The samples are loaded in 7 N HCl on a 1.6 mL AG-MP1 resin. The resin is further washed by passing 8 mL of 7 N HCl and the Cu is collected in 22 mL of 7 N HCl. This procedure is reproduced twice to ensure a clean Cu fraction. Copper isotopic data were measured using the MC-ICP-MS (Thermo-Finnigan Neptune Plus) located at IPGP following the method used in Savage *et al*.^31^. Briefly, copper is injected using a glass spray chamber and a 100 μL/min ESI Teflon micro nebulizer. The MC-ICP-MS was operated in low resolution mode with ^65^Cu and ^63^Cu collected in the central and L2 faraday cups, respectively.

The sample dilutions were adjusted (to within ~ ±5%) to match the concentration of the standard. The total yield of Cu is >99%. The blank of the full procedure is ~5 ng which is negligible in comparison to the amount of Cu (>200 ng) present in each samples. Typical external reproducibility estimated from numerous replicates was estimated to be better than 50 ppm (2 standard deviation)^31^.

The isotopic composition of Cu is reported as parts per 1,000 deviations relative to the NIST SRM 976 standard^16^.:

1 $${{\rm{\delta }}}^{65}{\rm{Cu}}=[\frac{{(\frac{{}^{65}{\rm{C}}{\rm{u}}}{{}^{63}{\rm{C}}{\rm{u}}})}_{{\rm{sample}}}}{{(\frac{{}^{65}{\rm{C}}{\rm{u}}}{{}^{63}{\rm{C}}{\rm{u}}})}_{{\rm{SRM}}-976}}-1]\times 1000$$

We used the standard bracketing method that consists of measuring a standard before and after each sample and use the average of the two standards for the normalizing ratio^16^. During analysis session the standard utilized was IPGP-Cu and relative to NIST SRM 976, the Cu_IPGP standard has δ^65^Cu = +0.271 ± 0.006‰ (2sd; n = 55). All the data presented here were converted to δ^65^Cu relative to NIST SRM 976 by adding 0.271.

### Statistical analysis

Statistical analysis was performed using GraphPad Prism. Data are represented as box-and-whiskers graphs, with the box always extending from the 25th to 75th percentiles, the line representing the median, and the whiskers showing the minimal and maximal values. Mann Whitney non-parametric tests were used to compare two groups of unpaired data sets. Two-way ANOVAs were performed to test the effect of various factors (age, sex and genotype), followed by Sidak’s multiple comparison test, or Wilcoxon matched-pairs signed rank test for paired data sets.

## Results

### Distinct copper isotopic composition of serum and brain in wild-type mice

We measured the Cu isotopic data for 75 serum and 9 brain samples, reported in Table S1. The serum samples represent nineteen 3-month-old, twenty-three 6-month-old, twenty 9-month-old and thirteen 12-month-old mice. The nine brains have been taken from 12-month-old mice. The average of the different groups of samples sorted by ages and sex are reported in Table 1.

**Table 1:** Average isotopic composition of brain and serum samples. Numbers indicated correspond to mean +/− SD of δ^65^Cu (number of mice). nd, not determined.

		Serum 3 months	Serum 6 months	Serum 9 months	Serum 12 months	Brain 12 months
Wild type	Males	−0.74 +/− 0.09 (n = 4)	−0.74 +/− 0.22 (n = 5)	−0.89 +/− 0.09 (n = 4)	nd (n = 0)	nd (n = 0)
	Females	−0.51 +/− 0.11 (n = 5)	−0.48 +/− 0.23 (n = 5)	−0.54 +/− 0.23 (n = 5)	−0.31 +/− 0.19 (n = 5)	0.63 +/− 0.26 (n = 5)
APPswe/ PSEN1dE9	Males	−0.78 +/− 0.23 (n = 5)	−0.76 +/− 0.04 (n = 7)	−0.70 +/− 0.19 (n = 7)	−0.65 +/− 0.08 (n = 3)	nd (n = 0)
	Females	−0.49 +/− 0.25 (n = 5)	−0.53 +/− 0.20 (n = 6)	−0.56 +/− 0.23 (n = 4)	−0.59 +/− 0.28 (n = 5)	0.51 +/− 0.15 (n = 5)

**Figure 1:**
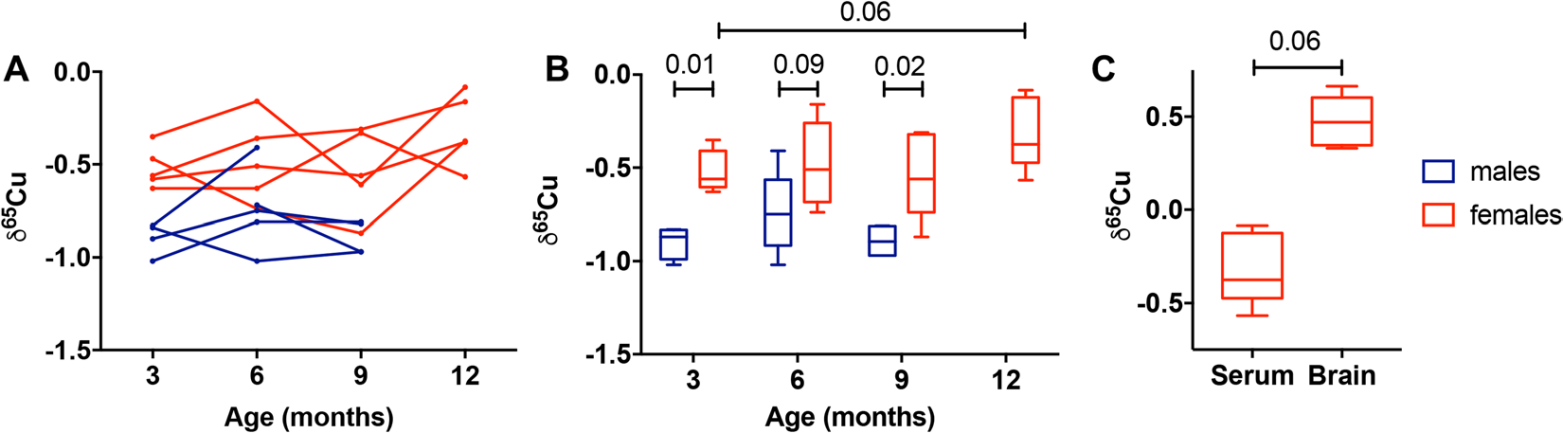
Copper isotopic composition of serum and brain in wild type mice. The data are reported as δ^65^Cu, which is the per mil deviation of the ^65^Cu/^63^Cu from the NIST 976 standard. (**A**) Evolution of δ^65^Cu in serum of individual mice are shown over time (males are shown in blue and female in red). (**B**) Pooled data for δ^65^Cu in serum: boxes extend from the 25^th^ and 75^th^ percentile, the line in the middle of the box represents the median, and the whiskers show the minimum and the maximum. Two-way ANOVA showed a significant effect of sex (p < 0001). p values between males and females are indicated on the graphic for each age (Sidak’s multiple comparison test). Wilcoxon matched-pairs rank test were used to compare females of different ages (p = 0.06 when comparing 3- and 12-month-old females). (**C**) Comparison of δ^65^Cu in serum and brain from 12-month-old females. p = 0.06, Wilcoxon matched-pairs rank test.

For WT mice, males (δ^65^Cu = −0.84 ± 0.16, sd) have isotopically lighter serum than females (δ^65^Cu = 0.44 ± 0.20, sd); the difference is statistically significant (p < 0.0001, Mann Whitney test). This difference is observed at every time point (Fig. 1A,B). The 3-, 6-, and 9-month-old mice have a constant δ^65^Cu value around −1.1 for males and −0.8 for females. Interestingly, the 12-month old females have a heavier serum isotopic composition (δ^65^Cu of −0.31 ± 0.19, sd; p = 0.06, Wilcoxon matched-pairs rank test).

**Figure 2:**
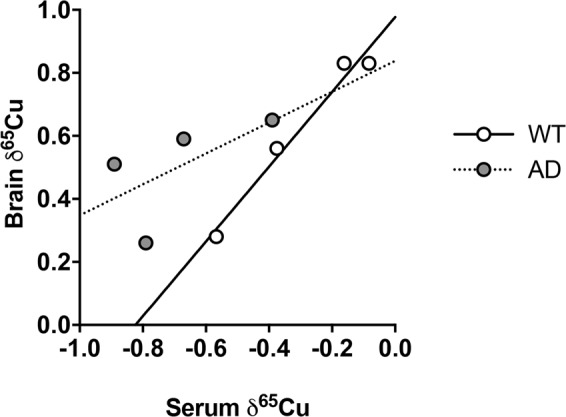
Correlation of the Cu isotopic composition between brain and serum. Data show δ^65^Cu values from serum of WT and AD 12-month-old female mice. Linear regression showed a significant positive correlation between serum and brain δ^65^Cu values for WT animals (r^2^ = 0.97; slope 1.19 ± 0.14, se, p = 0.01), but only a trend for AD animals (r^2^ = 0.62; slope 0.49 ± 0.44, se, p = 0.38).

We next compared the isotopic composition of brain and serum in 12-month-old females. Brains (δ^65^Cu = 0.63 ± 0.26, sd) are ~1‰ isotopically heavier than serum (p = 0.06, Wilcoxon matched-pairs rank test). Interestingly, the δ^65^Cu of the brains and associated serum are positively correlated (Figs 1C, 2, r^2^ = 0.97, slope statistically different from 0 as p = 0.01).

Old AD mice have a slightly lighter (but not statistically different) copper isotopic composition in their serum and brain.

**Figure 3:**
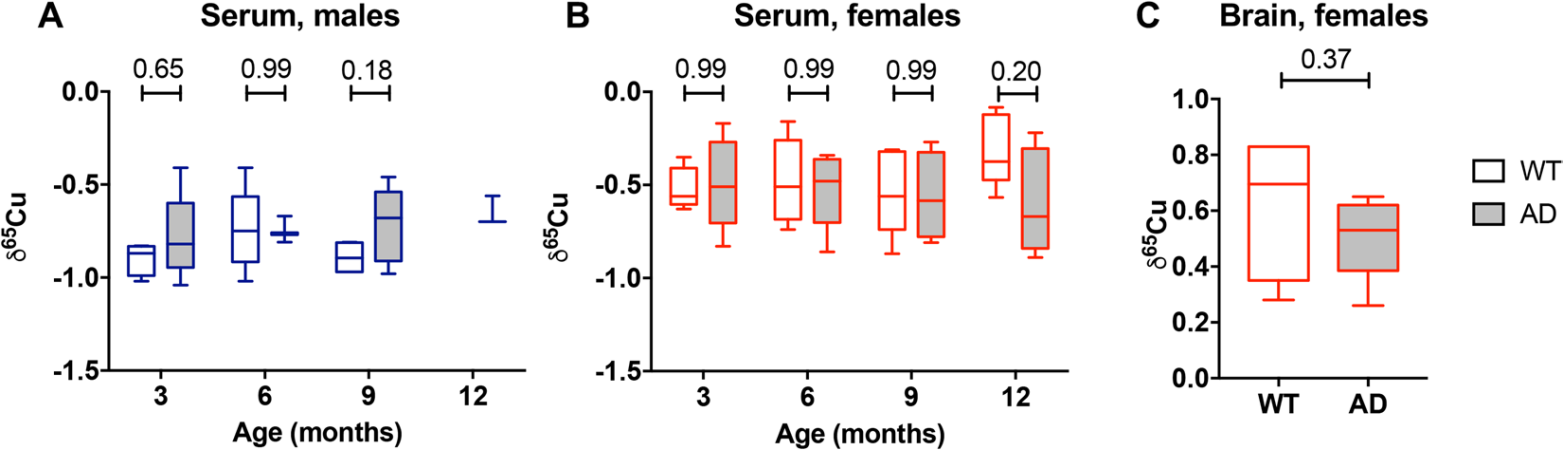
Effect of AD on Cu isotopic composition. (**A**,**B**). Data show δ^65^Cu values from serum of WT and AD mice. Two-way ANOVA showed no significant effect of age or genotype for serum in males (**A**) or females (**B**). p values between WT and AD are indicated on the graphic for each age (Sidak’s multiple comparison test). (**C**) δ^65^Cu in the brain of 12-month-old females. p = 0.37, Mann-Whitney test.

We investigated if AD would lead to a different copper isotopic composition in serum and/or brain by analyzing the isotopic composition of organs from the APPswe/PSEN1dE9 transgenic mouse model. The serum of 12-month-old AD females (δ^65^Cu = −0.59 ± 0.28, sd) was isotopically lighter than the serum of age-matched WT female littermates (δ^65^Cu of −0.31 ± 0.19, sd), even if the difference is not significant (p = 0.20, Sidak’s multiple comparison test) (Fig. 3B). There was no other significant difference at any age between WT and AD mice in serum. Similarly, the brains of the 12-month-old AD females (δ^65^Cu = 0.51 ± 0.15, sd) also appear modestly lighter (but not statistically significant) than the brain of the female WT littermate (δ^65^Cu = 0.63 ± 0.26, sd; see Fig. 3C).

## Discussion

### Isotopic difference between brain and serum

Our observation that brains (9 samples) are typically 1‰ heavier than serum (74 samples) in mice is consistent with previous data obtained on a smaller sample pool of samples. Balter *et al*.^13^ reported 4 brain samples and 9 whole blood samples from 5-month-old mice with an isotopic difference of ~0.8‰. On the other hand, human serum are heavier than what is reported here for mice by ~0.5‰ (e.g. refs^32–35^).

The differences of Zn isotopic composition between organs were previously attributed to the difference of bonding environment^12,13^. In mice, Zn is isotopically lighter in brain than in serum, which is explained by the fact that Zn is principally bound to cysteine-rich protein in the brain (metallothionein) while it is bound to histidine-rich protein in the serum (albumin)^36^. Copper shows the reverse isotopic effect (brain heavier than serum). The bonding properties and the associated isotopic fractionation is more complex in the case of Cu than for Zn because of the multiple redox state of Cu (I and II). Copper is principally found as Cu(I) in metallothionein and glutathione in the brain^23^ and as Cu(II) in synapses^24^. In human’s serum, Cu is mainly bound to ceruloplasmin (~90%; ref.^37^) with the rest being bound to β-macroglobulin, albumin, and in some small molecules^37–39^. In ceruloplasmin, Cu is principally found under its +II form and bounds to cysteine and histidine^40^. It is also found under its +II in β-macroglobulin^41^, while in albumin it could be under the +I form^42^. Since the majority of Cu in the brain should be stored under its isotopically light reduced form in metallothionein or glutathione^22,23^ while it should be mostly found as its isotopically heavy oxidised form in ceruloplasmin in the serum, the clearly isotopically heavier brain is surprising. In the absence of quantitative study on the speciation of Cu in the brain it is not possible to conclude on this topic here, but one possible explanation to this conundrum is that Cu(II) involved in synaptic transmission^24^ represent a large fraction of the total Cu stored in the brain.

The observation that serum in human (e.g. ref.^35^) is isotopically heavier than in mouse by ~0.5‰ suggests that the speciation of Cu differs in mice compare to humans. This is supported by previous HPLC-ICPMS measurements on plasma doped with ^65^Cu^37^ who found that ceruloplasmin is a more important ligand in human’s plasma compared to mice’s plasma. We can therefore conclude that Cu speciation in mouse serum is different than in human. Therefore, it will be important to test other AD animal models (e.g. minipigs^43^) as well as human patients.

The correlation that is observed between the δ^65^Cu of the brain and serum of 12-month old mice (Fig. 2) suggests that the Cu from which we analysed the isotopic composition in the serum has been interacting with the brain and therefore must be mobile. Copper bound to ceruloplasmin is not mobile, as it was demonstrated that Cu homesotatis is unchanged in ceruloplasmin-deficient mice compared to WT^44^. This implies that ceruloplasmin does not play a role in the transport and transfer of Cu between serum and organs. Therefore, low molecular weight molecules must carry the mobile Cu fraction. This is concordant with the fact that the main Cu transporter, CRT1, bounds both Cu(I) and Cu(II) and can bound large fraction of Cu in the serum^45^.

### Effect of sex

The systematic difference of ~0.4‰ of the δ^65^Cu between WT male and female mice suggests an effect of sex on the Cu isotopic budget of the serum. Such a sex difference has been previously observed for Cu concentration in mouse plasma^46^. Our data represent the first example of a significant isotopic difference for mice of different sex, even if the limited data from previous work on Cu isotopes in mice hinted that there was a heavy isotope enrichment in the blood of females compared to males (~0.15‰; ref.^13^, n = 5 for the females and 4 for the males, compared to n = 19 and n = 13, respectively, in the present study). Such an effect of sex was not observed for Zn isotopes in mice^12,13,15,47^.

In human, a difference between sexes was previously reported for Fe and Cu isotopes^47–49^. A difference of ~0.20‰ in Cu isotopic composition was also previously seen between men and premenopausal women in the whole blood (−0.52 vs −0.74)^50^. The origin of the Cu isotopic fractionation between men and premenopausal women is usually attributed to the larger Cu turnover in women due to menstruations (e.g. refs^51,52^). Such origin is not applicable to male and female mice since the reproductive cycle of mice does not include bleeding^53^. The sex isotopic difference in mice may be related to a difference of Cu speciation and relative abundance of Cu carrier in serum. However, to our knowledge, there is no study on the effect of sex on the speciation of Cu in mouse blood.

### Effect of AD on the Cu isotopic composition of the brain and serum

Twelve-month old AAPswe/PSEN1de9 female mice have slightly isotopically lighter brains in average than WT, even if this difference is not statistically significant. Isotope fractionation in 12-month old AAPswe/PSEN1de9 mice was previsouly observed for Zn^15^. Aβ plaques starts to develop in AAPswe/PSEN1de9 mice after 6 months with profuse plaques in the hippocampus and cortex at nine months^27^ and a proliferation until 12 months^28^. The small enrichment in light Cu isotopes of 12-month AAPswe/PSEN1de9 mice brains compared to WT suggests that the change of speciation associated with the proliferation of Aβ plaques modifies the global Cu isotopic composition of the brain. It is known that the formation of Aβ plaques increases the production of free Cu+ in the brain and therefore induces oxidative damages^26^. Such increase in Cu+ in the brain should globally decrease the δ^65^Cu value of the whole brain. On the other hand, copper is primarily bound to metallothionein in normal brain while in Aβ plaques it is bound to histidine^54,55^. Metallothionein is a cysteine rich protein and at 298 K it has a logarithm of the reduced partition function (lnβ) of ~3.3 (refs^16,56^) while in the same conditions lnβ of histidine is ~4.4. As a result, Aβ plaques should be isotopically heavier than normal brain. However, it was also shown that Aβ has a bounding site that favors Cu(I)^20^, which would conversely enrich the Aβ in the light isotope of Cu. If confirmed, the enrichment in the light isotopes of the AD mouse brains would imply that the production and increase of free Cu+ ion in the brain globally, together with the bounding of Cu(I) to Aβ, decrease the ^65^Cu/^63^Cu ratio.

Similarly to the brain, the Cu isotopic composition of the serum of the 12-month old AAPswe/PSEN1de9 mice tends to be isotopically different from the WT mice (but again statistically not different), while it is unchanged from 3 to 9 month in both the WT and AD mice. It is enriched in the lighter isotopes compared to WT mouse serum, such that the δ^65^Cu value in the serum and in the brain correlate (Fig. 2). This correlation between the δ^65^Cu value of the brain and the serum of the 12-month-old mice suggests that the change of composition of the serum is linked to the change happening in the brain. It was actually shown that the Cu level of the serum is higher in AD patients than in controls^25,57^. In particular, it was shown that AD serum has an increase of non ceruloplasmin Cu. An increase of Cu(I) species would decrease the total δ^65^Cu value of the serum. The origin of this non ceruloplasmin Cu was actually suggested to be a transfer of Cu(I) from the brain to the serum^20,58^ which would explain our observations.

## Conclusion

Copper isotopic composition of serum is a potential tool to study the change of homeostasis associated with AD. Here we show that the δ^65^Cu value of WT males is ~0.4 different from WT females. This suggests an effect of sex on the Cu isotopic budget of the serum, which may be related to a difference of Cu speciation and relative abundance of Cu carrier in serum. We also find a trend that suggests that Aβ plaques induced a change in the Cu isotopic composition of the whole brain and of the serum. This tendency could be explained by the increase of isotopically light Cu(I) species in the brain. The Cu isotopic composition of the brain and of the serum correlate, which can be explained by a transfer of Cu(I) species from the brain to the serum and suggest that monitoring the natural Cu isotopic ratios in the serum may be used to detect the formation of Aβ plaques in the brain, but further work will have to be done to explore this hypothesis. The difference of speciation of Cu in the serum of mice compared to human calls for exploring different animal models (e.g. minipigs) and humans.

## Supplementary information

Table S1

## Data Availability

All data are available in supplementary materials.

## References

[1] Mortality GBD, Causes of Death C (2015). Global, regional, and national age-sex specific all-cause and cause-specific mortality for 240 causes of death, 1990–2013: a systematic analysis for the Global Burden of Disease Study 2013. Lancet.

[2] Shaw LM (2009). Cerebrospinal Fluid Biomarker Signature in Alzheimer’s Disease Neuroimaging Initiative Subjects. Ann. Neurol..

[3] Mattsson N (2009). CSF Biomarkers and Incipient Alzheimer Disease in Patients With Mild Cognitive Impairment. JAMA-J. Am. Med. Assoc..

[4] Villemagne VL (2011). Longitudinal Assessment of A beta and Cognition in Aging and Alzheimer Disease. Ann. Neurol..

[5] Baum L (2010). Serum zinc is decreased in Alzheimer’s disease and serum arsenic correlates positively with cognitive ability. Biometals.

[6] Miller LM (2006). Synchrotron-based infrared and X-ray imaging shows focalized accumulation of Cu and Zn co-localized with beta-amyloid deposits in Alzheimer’s disease. J. Struct. Biol..

[7] Religa D (2006). Elevated cortical zinc in Alzheimer disease. Neurology.

[8] Rulon LL (2000). Serum zinc levels and Alzheimer’s disease. Biological Trace Element Research.

[9] Haines A, Iliffe S, Morgan P, Dormandy T, Wood B (1991). Serum alumnium and zin and other variables in patients ith and without cognitive impairment in the community. Clin Chim Acta.

[10] Vasto S (2007). Zinc and inflammatory/immune response in aging. Ann N Y Acad Sci..

[11] Balter V (2010). Bodily variability of zinc natural isotope abundances in sheep. Rapid Commun Mass Spectrom.

[12] Moynier F, Fujii T, Shaw A, Le Borgne M (2013). Heterogeneous distribution of natural zinc isotopes in mice. Metallomics.

[13] Balter V (2013). Contrasting Cu, Fe, and Zn isotopic patterns in organs and body fluids of mice and sheep, with emphasis on cellular fractionation. Metallomics.

[14] Faller, P. & Hureau, C. Bioinorganic chemistry of copper and zinc ions coordinated to amyloid-beta peptide. *Dalton Trans*, 1080–1094, 10.1039/b813398k (2009). 10.1039/b813398k 19322475

[15] Moynier F, Foriel J, Shaw A, Le Borgne M (2017). Zinc isotopic behavior during Alzheimer’s disease. Geochemical Perspective Letters.

[16] Moynier, F., Vance, D., Fujii, T. & Savage, P. In Non-traditional stable isotopes Vol. 82 (eds Teng, F-Z, Watkins, J. & Dauphas, N.) 543–600 (Mineralogical Society of America, 2017).

[17] Sauzeat L (2018). Isotopic Evidence for Disrupted Copper Metabolism in Amyotrophic Lateral Sclerosis. iScience.

[18] Rajendran R (2009). A novel approach to the identification and quantitative elemental analysis of amyloid deposits–insights into the pathology of Alzheimer’s disease. Biochemical and biophysical research communications.

[19] Lovell MA, Robertson JD, Teesdale WJ, Campbell JL, Markesbery WR (1998). Copper, iron and zinc in Alzheimer’s disease senile plaques. Journal of the neurological sciences.

[20] Barnham KJ (2003). Structure of the Alzheimer’s disease amyloid precursor protein copper binding domain. A regulator of neuronal copper homeostasis. J Biol Chem.

[21] Syme C, Nadal R, Rygby S, Viles J (2004). Copper Binding to the Amyloid-β (Aβ) Peptide Associated with Alzheimer’s Disease. J Biol Chem.

[22] Vašák M, Meloni G (2017). Mammalian Metallothionein-3: New Functional and Structural Insights. Int. J. Mol. Sci..

[23] Scheiber I, Mercer J, Dringen R (2014). Metabolism and functions of copper in brain. Progress in neurobiology.

[24] Gaier ED, Eipper BA, Mains RE (2013). Copper signaling in the mammalian nervous system: synaptic effects. J Neurosci Res.

[25] Squitti R (2005). Excess of serum copper not related to ceruloplasmin in Alzheimer disease. Neurology.

[26] Barnham KJ, Masters CL, Bush AI (2004). Neurodegenerative diseases and oxidative stress. Nature reviews. Drug discovery.

[27] Jankowsky JL (2004). Mutant presenilins specifically elevate the levels of the 42 residue beta-amyloid peptide *in vivo*: evidence for augmentation of a 42-specific gamma secretase. Hum Mol Genet.

[28] Garcia-Alloza M (2006). Characterization of amyloid deposition in the APPswe/PS1dE9 mouse model of Alzheimer disease. Neurobiol Dis.

[29] Maréchal C, Télouk P, Albarède F (1999). Precise analysis of copper and zinc isotopic compositions by plasma-source mass spectrometry. Chem. Geol..

[30] Rodovka Z (2017). Zinc and copper isotope systematics in sediments from the Ries Impact Structure and central European tektites – implications for material sources and loss of volatiles. Meteorit. Planet. Sci..

[31] Savage P (2015). Copper isotope evidence for large-scale sulphide fractionation during Earth’s differentiation. Geochemical Perspective Letters.

[32] Telouk P (2015). Copper isotope effect in serum of cancer patients. A pilot study. Metallomics.

[33] Costas-Rodriguez M (2015). Isotopic analysis of Cu in blood serum by multi-collector ICP-mass spectrometry: a new approach for the diagnosis and prognosis of liver cirrhosis?. Metallomics.

[34] Lauwens S, Costas-Rodriguez M, Van Vlierberghe H, Vanhaecke F (2016). Cu isotopic signature in blood serum of liver transplant patients: a follow-up study. Sci Rep.

[35] Balter V (2015). Natural variations of copper and sulfur stable isotopes in blood of hepatocellular carcinoma patients. Proc Natl Acad Sci USA.

[36] Scott BJ, Bradwell AR (1983). Identification of the Serum Binding-Proteins for Iron, Zinc, Cadmium, Nickel, and Calcium. Clin Chem.

[37] Cabrera A (2008). Copper binding components of blood plasma and organs, and their responses to influx of large doses of (65)Cu, in the mouse. Biometals.

[38] Cousins RJ (1985). Absorption, Transport, and Hepatic-Metabolism of Copper and Zinc - Special Reference to Metallothionein and Ceruloplasmin. Physiol Rev.

[39] Wirth PL, Linder M (1985). Distribution of copper among components of human serum. J. Natl. Cancer Inst.

[40] Bento I, Peixoto C, Zaitsev VN, Lindley PF (2007). Ceruloplasmin revisited: structural and functional roles of various metal cation-binding sites. Acta crystallographica. Section D, Biological crystallography.

[41] Moriya M (2008). Copper is taken up efficiently from albumin and alpha2-macroglobulin by cultured human cells by more than one mechanism. American journal of physiology. Cell physiology.

[42] Sendzik, M., Pushie, J., Stefaniak, E. & Haas, K. Structure and Affinity of Cu(I) Bound to Human Serum Albumin. *Inorg. Chem*. **56**, 15057–15065. 10.1021/acs.inorgchem.7b02397 29166002

[43] Mahan B, Moynier F, Jorgensen AL, Habekost M, Siebert J (2018). Examining the homeostatic distribution of metals and Zn isotopes in Gottingen minipigs. Metallomics.

[44] Meyer LA, Durley AP, Prohaska JR, Harris ZL (2001). Copper transport and metabolism are normal in aceruloplasminemic mice. J Biol Chem.

[45] Lutsenko S (2016). Copper trafficking to the secretory pathway. Metallomics.

[46] Quinn JF (2011). Gender effects on plasma and brain copper. Int J Alzheimers Dis.

[47] Albarede F, Telouk P, Lamboux A, Jaouen K, Balter V (2011). Isotopic evidence of unaccounted for Fe and Cu erythropoietic pathways. Metallomics.

[48] Walczyk T, von Blanckenburg F (2005). Deciphering the iron isotope message of the human body. Int. J. Mass spectrom..

[49] Jaouen K (2012). Fe and Cu stable isotopes in archeological human bones and their relationship to sex. Am J Phys Anthropol.

[50] Jaouen K (2013). Is aging recorded in blood Cu and Zn isotope compositions?. Metallomics.

[51] Van Heghe L, Deltombe O, Delanghe J, Depypere H, Vanhaecke F (2014). The influence of menstrual blood loss and age on the isotopic composition of Cu, Fe and Zn in human whole blood. J. Anal. At. Spectrom..

[52] Jaouen K, Balter V (2014). Menopause effect on blood Fe and Cu isotope compositions. Am J Phys Anthropol.

[53] Byers SL, Wiles MV, Dunn SL, Taft RA (2012). Mouse estrous cycle identification tool and images. Plos One.

[54] Tougu V, Karafin A, Palumaa P (2008). Binding of zinc(II) and copper(II) to the full-length Alzheimer’s amyloid-beta peptide. Journal of neurochemistry.

[55] Karr JW, Akintoye H, Kaupp LJ, Szalai VA (2005). N-Terminal deletions modify the Cu2+ binding site in amyloid-beta. Biochemistry-Us.

[56] Fujii T, Moynier F, Blichert-Toft J, Albarede F (2014). Density functional theory estimation of isotope fractionation of Fe, Ni, Cu, and Zn among species relevant to geochemical and biological environments. Geochim. Cosmochim. Acta.

[57] Squitti R (2002). Elevation of serum copper levels in Alzheimer’s disease. Neurology.

[58] Squitti R (2006). Excess of nonceruloplasmin serum copper in AD correlates with MMSE, CSF [beta]-amyloid, and h-tau. Neurology.

