# Supplementary material for: Serum and brain natural copper stable isotopes in a mouse model of Alzheimer’s disease
Source: Sci Rep. 2019 Aug 15;9:11894. doi: 10.1038/s41598-019-47790-5 (PMC6695409; doi:10.1038/s41598-019-47790-5)
Supplement: Supplementary file 1 — Table S1 [file 41598_2019_47790_MOESM1_ESM.pdf]

Serum and brain natural copper stable isotopes in a mouse model of Alzheimer's disease

Frédéric Moynier[1,2], John Creech[1], Jessica Dallas[1], and Marie Le Borgne[3]

[1]Institut de Physique du Globe de Paris, Université de Paris, CNRS, 1 rue Jussieu 75238 Paris cedex 05 France

[2]Institut Universitaire de France, 75005, Paris, France

[3] Unité 1148, Institut National de la Santé et de la Recherche Médicale (INSERM), Hôpital Xavier Bichat, Paris, France; Université Paris Diderot, Sorbonne Paris Cité, Paris, France; Département Hospitalo-Universitaire DHU FIRE, Paris, France

Corresponding author: Frédéric Moynier, moynier@ipgp.fr

Other authors email addresses:

john@isospike.org

dallas@ipgp.fr

marie.le-borgne-moynier@inserm.fr

			3 months - Serum			6 months - Serum			9 months - Serum			12 months - Serum			12 months - Brain		
Mouse ID	Genotype	Sex	$\delta^{65}Cu$ (vs NIST SRM-976)	±	n	$\delta^{65}Cu$ (vs NIST SRM-976)	±	n	$\delta^{65}Cu$ (vs NIST SRM-976)	±	n	$\delta^{65}Cu$ (vs NIST SRM-976)	±	n	$\delta^{65}Cu$ (vs NIST SRM-976)	±	n
10	WT	female	-0.63	0.03	1	-0.63	0.03	1	-0.33	0.03	1	-0.57	0.03	3	0.28	0.00	3
13	WT	female	-0.58	0.02	1	-0.51	0.04	1	-0.56	0.03	1	-0.38	0.04	2			
14	WT	female	-0.56	0.02	1	-0.36	0.04	1	-0.31	0.02	1	-0.16	0.02	2	0.83	0.03	3
16	WT	female	-0.35	0.02	1	-0.16	0.05	1	-0.61	0.03	1	-0.08	0.12	2	0.83	0.04	1
29	WT	female	-0.47	0.07	1	-0.74	0.03	1	-0.87	0.02	1	-0.38	0.05	2	0.56	0.03	1
2	WT	male	-0.90	0.02	1	-0.75	0.09	2	-0.82	0.03	1						
4	WT	male	-1.02	0.02	1	-0.81	0.03	1	-0.81	0.02	1						
21	WT	male	-0.84	0.03	1	-1.02	0.04	1	-0.97	0.03	1						
24	WT	male	-0.83	0.02	1	-0.41	0.04	1									
26	WT	male				-0.72	0.03	1	-0.97	0.04	1						
9	AD	female	-0.83	0.03	1	-0.86	0.01	1	-0.81	0.04	1	-0.79	0.00	2	0.26	0.05	3
11	AD	female				-0.37	0.05	1	-0.27	0.02	1	-0.22	0.04	2			
12	AD	female	-0.37	0.02	1	-0.34	0.05	1				-0.39	0.23	2	0.65	0.04	3
15	AD	female	-0.58	0.03	1	-0.38	0.04	1	-0.68	0.03	1	-0.67	0.02	1	0.59	0.03	1
17	AD	female	-0.51	0.02	1	-0.65	0.03	1	-0.76	0.03	1				0.53	0.06	1
31	AD	female	-0.17	0.02	1	-0.58	0.02	1				-0.89	0.14	2	0.51	0.01	1
1	AD	male	-0.85	0.03	1	-0.76	0.07	2	-0.72	0.05	1						
3	AD	male	-0.82	0.04	1	-0.77	0.02	1	-0.68	0.03	1						
5	AD	male	-1.04	0.02	1	-0.67	0.02	1	-0.46	0.04	1						
6	AD	male				-0.76	0.03	1	-0.59	0.02	1	-0.70	0.02	1			
7	AD	male				-0.76	0.03	1	-0.54	0.04	1	-0.70	0.01	2			
20	AD	male	-0.79	0.02	1	-0.76	0.04	1	-0.98	0.02	1	-0.56	0.08	2			
25	AD	male	-0.41	0.02	1	-0.81	0.03	1	-0.91	0.03	1						

Table S1. Copper isotopic composition of brain and serum samples.

n=number of measurements. WT=wild type mice, AD=APPswe/PSEN1dE9 mice.
